# Unraveling the role of dark septate endophyte (DSE) colonizing maize (*Zea mays*) under cadmium stress: physiological, cytological and genic aspects

**DOI:** 10.1038/srep22028

**Published:** 2016-02-25

**Authors:** Jun-ling Wang, Tao Li, Gao-yuan Liu, Joshua M. Smith, Zhi-wei Zhao

**Affiliations:** 1 State Key Laboratory of Conservation and Utilization for Bioresources in Yunnan, Yunnan University, Kunming, 650091 Yunnan, P.R. China; 2 Key Laboratory of Microbial Diversity in Southwest China, Ministry of Education, Yunnan University, Kunming, 650091 Yunnan, P.R. China; 3 First People’s Hospital of Qujing City, Qujing Affiliated Hospital of Kunming Medical University, Qujing 655000, China; 4 Irving K. Barber School of Arts and Sciences, University of British Columbia Okanagan, Kelowna, British Columbia V1V 1V7, Canada

## Abstract

A growing body of evidence suggests that plant root-associated fungi such as dark septate endophytes (DSE) can help plants overcome many biotic and abiotic stresses, of great interest is DSE-plant metal tolerance and alleviation capabilities on contaminated soils. However, the tolerance and alleviation mechanisms involved have not yet been elucidated. In the current study, the regulation and physiological response of *Zea mays* to its root-associated DSE, *Exophiala pisciphila* was analyzed under increased soil Cd stress (0, 10, 50, 100 mg kg^−1^). Under Cd stress, DSE inoculation significantly enhanced the activities of antioxidant enzymes and low-molecular weight antioxidants, while also inducing increased Cd accumulation in the cell wall and conversion of Cd into inactive forms by shoot and root specific regulation of genes related to metal uptake, translocation and chelation. Our results showed that DSE colonization resulted in a marked tolerance to Cd, with a significant decrease in cadmium phytotoxicity and a significant increase in maize growth by triggering antioxidant systems, altering metal chemical forms into inactive Cd, and repartitioning subcellular Cd into the cell wall. These results provide comprehensive evidence for the mechanisms by which DSE colonization bioaugments Cd tolerance in maize at physiological, cytological and molecular levels.

Heavy metal contamination of soils is a serious global environmental issue. Millions of hectares of arable land in conventional agriculture regions worldwide, for example about 3.2 Mha in China, are affected by the presence of elevated amounts of plant-available heavy metals, causing direct economic losses, reduction in food and feed quality and safety concerns^1^,^2^. Considering that contaminated soils are still a potential resource for agricultural production, a novel soil remediation strategy known as phytomanagement has recently received a lot of attention^3^. Phytomanagement can combine profitable crop production with the gradual reduction of soil contamination by phytoextraction, and this makes it attractive and beneficial to farmers, industry and regulators^4^. For phytomanagement purposes, energy maize (*Zea mays*) is widely used a phytoremediation crop due to its high biomass and low metal uptake capacity in moderately contaminated soils on which conventional agriculture is impaired^5^. In Europe, production of energy maize, used for biogas production through anaerobic digestion, is increasing rapidly^6^,^7^. For safe production of these fast-growing-like crops, numerous approaches such as the selection of low-accumulating cultivars and the application of soil amendments e.g. sludge, biochar, compost, fertilizer have been developed^2^,^8^,^9^. Increasingly, data shows that the combined use of plants and their root-associated microorganisms, like plant growth-promoting rhizobacteria, mycorrhizal fungi, dark septate endophytes etc., are able to remove, contain, inactivate, or degrade harmful environmental contaminants (generally termed phytoremediation) and revitalize contaminated sites^2^,^10^,^11^,^12^,^13^,^14^,^15^.

Recent data has revealed that ubiquitous DSEs are essential components of soil microflora in metal-polluted ecosystems, and play multifunctional roles for plant growth and survival^12^,^16^,^17^. For example, field surveys from Ban, *et al.*^18^ showed that DSE colonization was negatively correlated with the translocation of Pb (r = −0.88, *p* < 0.05) accumulated in six dominant plant species grown on Qiandongshan Pb-Zn mine tailings, China. To evaluate the role of DSEs in phytoremediation of radioactive cesium (Cs), Diene, *et al.*^19^ identified the distinct responses of DSE-inoculation on Cs accumulation in two commercial crops; they observed that DSEs significantly decreased Cs accumulation in DSE-inoculated tomato but enhanced phytoremediation of Cs by Chinese cabbage, while also significantly increasing the biomass of both crops by up to 82 and 122% (for Chinese cabbage and tomato, respectively), compared to non-inoculated controls. Also, data from Likar and Regvar^14^ revealed that DSEs isolated from *Salix caprea* reduced metal uptake and improved the general physiology of their host plants via various effects, such as increasing chlorophyll concentrations and transpiration rates, for example. Our previous findings also showed that root colonization by *Exophiala pisciphila* significantly alleviated the deleterious effects of excessive heavy metals and promoted the maize growth under heavy-metal stress conditions^11^. However, actual knowledge on the physiological and cytological mechanisms by which DSEs function to alleviate the deleterious effects of excessive heavy metals on their host plants is still scarce, especially with regard to aspects of metal accumulation and distribution.

In the present study, *E. pisciphila* H93 was chosen as a model DSE-association to examine the cellular and enzymatic mechanisms of DSE-alleviated cadmium stress in *Zea mays*. The effect of DSE fungus on the growth and subcellular repartitioning of Cd in maize planted in Cd-contaminated soil was investigated. We also examined the chemical forms of Cd taken up by the plants, as well as the resulting quantity and chemical forms of Cd in various plant tissues. Our results will help to elucidate the interactions among DSE fungus, maize, and Cd-contaminated soil, and provide new insights into how DSE fungus may affect plant uptake and distribution of heavy metals.

## Results

### Influence of DSE colonization on Cd uptake of maize

**Figure 1:**
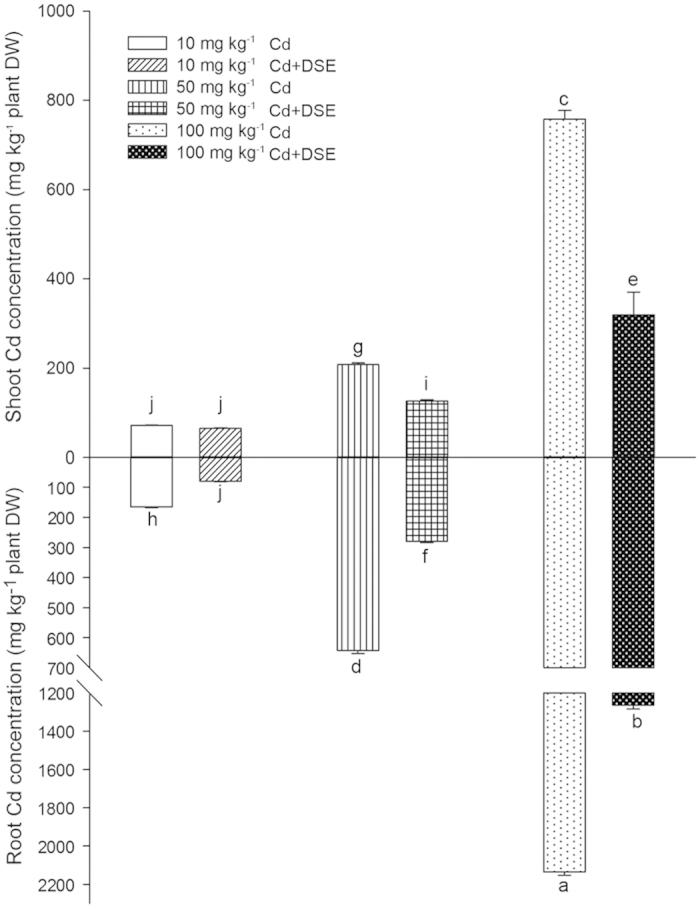
Shoot and root Cd concentrations (means ± SEM, *n* = 8) of maize with or without *E. pisciphila* H93 inoculation on culture substrate supplemented with 10, 50 or 100 mg kg^−1^ Cd^2+^. No Cd accumulated in maize under 0 mg kg^−1^ Cd^2+^ supplements (data not shown). Different letters on the bars indicate significant difference between the treatments (Tukey’s HSD, *p* < 0.05).

*E. pisciphila* H93 intensively colonized the roots of inoculated treatments with a range of 19% to 31% colonization after 30 days, and no DSE structures were observed in roots of non-inoculated treatments. Concentrations of accumulated Cd in the roots and shoots of all 8 treatments were assessed (Fig. 1). Both shoot and root Cd accumulation gradually increased with the elevated Cd supplements in the culture substrate for both inoculated and non-inoculated treatments; the majority of the Cd was absorbed and accumulated in root tissue compared to shoot tissue, regardless of inoculation treatment. However, the presence of *E. pisciphila* significantly decreased Cd concentrations in both shoots and roots compared to the non-inoculated maize in the medium and high Cd treatments. This was especially evident for Cd accumulation in maize roots, where significantly reduced Cd concentrations were observed at all Cd pollution levels (*p* < 0.001), compared with their respective non-inoculated controls. Additionally, the decrease in Cd concentrations in the root tissues of the medium and high-Cd treatments was also significantly higher than the reduction in Cd concentrations observed in the shoot tissues of the same metal-stressed treatments. These results indicate that the improved Cd tolerance of DSE-inoculated maize mainly resulted from both the reduced root Cd absorption and the constrained translocation of Cd from roots to shoots compared to the non-inoculated maize.

### Regulation of maize antioxidant systems by DSE to Cd exposure

**Figure 2:**
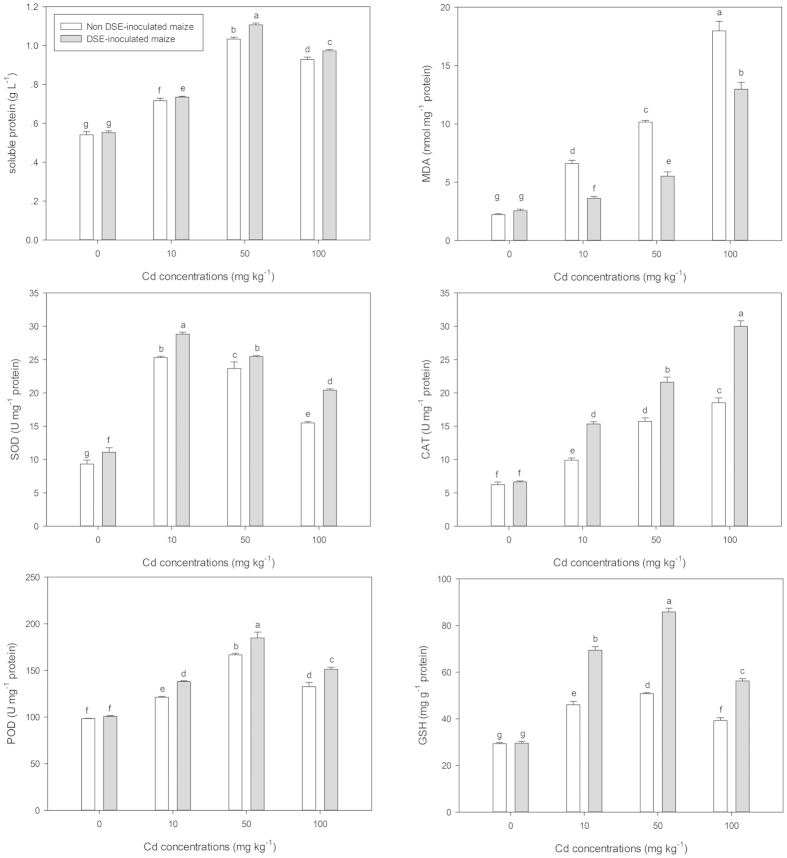
Effect of DSE inoculation on total soluble protein, MDA, SOD, CAT, POD and GSH concentrations in leaves of maize grown under different Cd stress concentrations (means ± SEM, *n* = 8). Different letters on the bars indicate significant difference between the treatments (Tukey’s HSD, *p* < 0.05).

The antioxidant systems of maize, including the activity of antioxidant enzymes and the concentration of antioxidant agents, as well as malondialdehyde (MDA), a reliable indicator of oxidative stress, were measured in the leaves via spectrophotometer (Fig. 2). Leaf MDA content was positively correlated with the Cd concentrations in the culture substrate, both for inoculated and non-inoculated treatments, but MDA content in DSE-inoculated maize grown in all Cd-stressed treatments was significantly reduced compared to the non-inoculated maize. Total leaf soluble proteins of both inoculated and non-inoculated maize significantly increased with the Cd amendments, with the highest concentration observed at 50 mg kg^−1^ Cd, followed by the 100 mg kg^−1^ Cd and then the 10 mg kg^−1^ Cd treatments. DSE-inoculation significantly increased the total soluble protein concentrations for all Cd treatment levels, compared to their respective non-inoculated controls. This trend was consistent across all ROS-scavenging antioxidant systems examined (SOD, CAT, POD and GSH), significantly increased concentrations were observed at all Cd treatment levels, independent of the presence or absence of DSE inoculation, with the DSE-inoculated treatments having significantly higher ROS antioxidant system activity than their respective non-inoculated controls. This was especially apparent for the activities of CAT observed, which is responsible for inactivating H_2_O_2_ into water. CAT activity increased with the elevated Cd concentrations in the culture substrate, with the maximum activity observed at the highest Cd^2+^ level tested (100 mg kg^−1^). However, the leaf SOD and POD activities and GSH concentrations reached peak at medium or low Cd stress, then decreased at the increased Cd concentrations. For example, SOD activity, responsible for the elimination of superoxide radicals in cells, increased significantly in the leaves of both DSE-inoculated and non-inoculated maize under the 10 mg kg^−1^ Cd treatment, yet declined at 50 and 100 mg kg^−1^ Cd compared to 10 mg kg^−1^ Cd; however, these values were still significantly higher than those of maize under no Cd supplements. Most importantly, root colonization by *E. pisciphila* greatly enhanced SOD, CAT and POD activities and GSH concentrations in the leaves of maize grown at all Cd treatments compared with those of non-inoculate maize. Under 100 mg kg^−1^ Cd-stress, the activities of leaf SOD, CAT, POD and GSH concentrations in DSE-inoculated maize increased 132%, 162%, 114% and 143%, respectively, compared to the non-inoculated controls, while MDA, a biomarker for oxidative stress, significantly reduced 72% due to DSE colonization.

### Subcellular distribution of Cd

**Figure 3:**
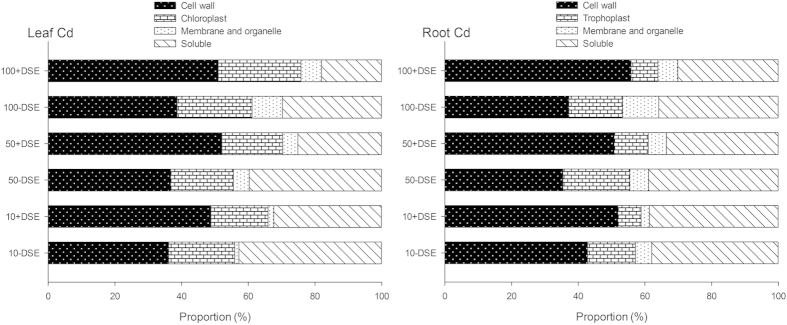
Proportional subcellular distribution of Cd in maize, inoculated with DSE or not, under a series of Cd stresses.

The subcellular distribution of Cd in maize leaves and roots was investigated for all treatments (Table 1, Fig. 3). Cd accumulation in each subcellular fraction was positively correlated to the increase in substrate Cd concentrations for both leaves and roots of maize, regardless of inoculation. Across all treatments, in both leaves and roots, maximum Cd accumulation always occurred in the cell wall, followed by the soluble fractions, and the minimum Cd accumulation was in the organelle fraction. Of note, Cd concentrations in all root or shoot subcellular fractions of maize (cell wall, chloroplast/trophoplast, membrane and organelle, and soluble) significantly decreased in the plants inoculated with DSE compared to the non-inoculated treatments, with the only observed exception to this statement being Cd concentration in the leaf cell wall of the 50 mg kg^−1^ treatments. Interestingly, DSE colonization increased the proportional Cd accumulation in cell wall, which resulted in significantly less soluble Cd in both leaves and roots (Fig. 3). For example, under 100 mg kg^−1^ Cd stress, 38.6% and 37.2% of Cd accumulation was in the leaf and root cell wall of the non-inoculated maize, compared to 51.0% and 55.8% of Cd accumulation in leaf and root cell wall of the DSE-inoculated plants. This led to a decrease in the proportional Cd accumulation in the soluble fraction from 29.8% and 35.9% to 18.0% and 30.1% with DSE inoculation, in leaves and roots respectively.

### Chemical forms of Cd

**Figure 4:**
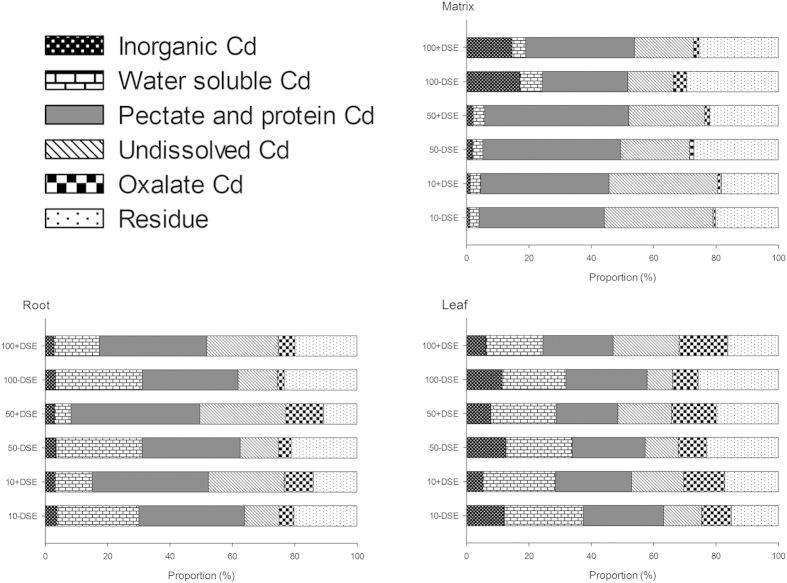
Proportions of Cd chemical forms found in maize leaves and roots, and the culture substrate inoculated with DSE or not, under a series of Cd stresses (0, 10, 50, 100 mg kg^−1^).

In the current study, silica sand was used as culture matrix and Cd concentrations of different chemical forms in the matrix and root and leaf tissues were assessed (Fig. 4, Table 2). After 30 days of growth, we found that Cd was primarily found as bound-pectate and protein, undissolved Cd or residue in the culture substrate of both inoculated and non-inoculated maize (Table 2). However, there was distinct compositional change in the chemical form of remaining substrate Cd in DSE-inoculated treatments compared to the non-inoculated treatments. DSE-inoculated culture substrate had significantly increased proportions of both undissolved Cd, and pectate and protein Cd in all Cd treatments, as well as significantly reduced ratios of residue Cd to total Cd (Fig. 4). There were also minor differences in the proportions of the other chemical Cd forms between DSE-inoculated and control group substrates.

In the present study, Cd concentrations in different chemical forms in maize leaves and roots increased with elevated Cd supply in the culture substrate (Table 2). For the roots, water soluble, undissolved, pectate and protein integrated and residue Cd were predominant in both non-inoculated and inoculated maize, whereas inorganic and oxalate Cd were less predominant. In contrast to the relatively consistent proportional Cd forms in maize roots, there was less consistency in the proportions of Cd forms in maize leaves for both the DSE-inoculated and the non-inoculated maize. Additionally, DSE colonization significantly changed the composition of Cd forms in both leaves and roots compared to the non-inoculated maize. The maize with DSE experienced decreased proportions of both water soluble Cd and residue Cd, while the ratios of both undissolved Cd and oxalate Cd increased, in both leaves and roots (Fig. 4 and Table 2). DSE inoculation also induced changes that were specific to tissue type; DSE-inoculated maize had a decreased proportion of pectate and protein integrated Cd in leaves but an increased proportion in the roots, compared to the non-inoculated controls. In contrast to the small variations observed in the proportion of root inorganic Cd in all treatments, the DSE maize leaf proportions of inorganic Cd were significantly reduced compared to non-inoculated maize (Fig. 4 and Table 2).

### Transcript levels of 3 key genes involved in Cd transport and detoxification

In the current study, three key genes encoding proteins involved in divalent cation uptake (*ZIP*), detoxification (*PCS*), and transport (*MTP*) were targeted in roots and leaves of maize, inoculated with DSE or not, exposed to 0, 10, 50, or 100 mg kg^−1^ Cd^2+^. In addition, a negative control qPCR was carried out to ensure that our maize qPCR data was not modified by DSE RNA in colonized roots and no PCR products were recorded; this suggested our maize qPCR data excluded interference from DSE RNA in colonized maize roots.

**Figure 5:**
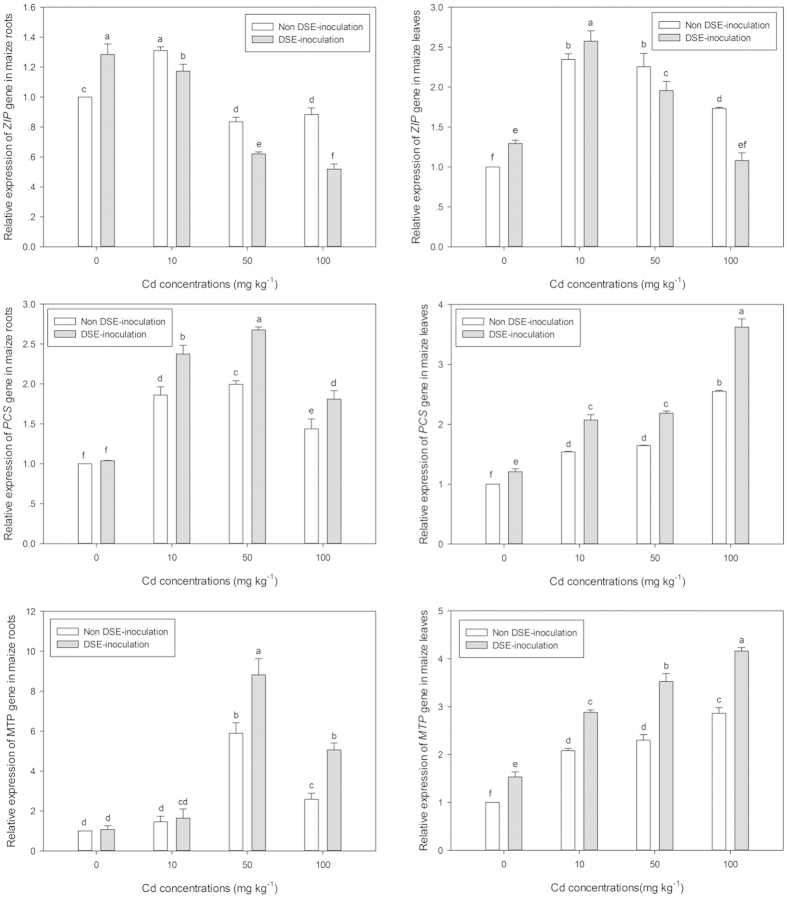
Expression patterns of three key genes encoding proteins involved in divalent cation uptake (*ZIP*), detoxification (*PCS*), transport (*MTP*) in roots and leaves of non-inoculated (-DSE) or inoculated (+DSE) maize exposed to 0, 10, 50, 100 mg kg^−1^ Cd^2+^ for 30 days. Different letters on the bars indicate significant difference between the treatments (Tukey’s HSD, *p* < 0.05).

qPCR data showed that *ZIP* expression in both leaves and roots reached peak under low Cd stress (10 mg kg^−1^) and then was down-regulated at higher Cd concentrations. In the 0 mg kg^−1^ Cd treatments, DSE colonization significantly up-regulated *ZIP* 1.28 times in roots and 1.29 times in leaves of maize, in contrast to non-inoculated maize. Under 10 mg kg^−1^ Cd stress, DSE inoculation mediated a distinct change in *ZIP* expression that differed between roots and their corresponding leaves; DSE colonization up-regulated leaf *ZIP* expression, yet resulted in *ZIP* down-regulation in maize roots, compared to non-inoculated plants. However, under the medium and high Cd concentration treatments, DSE-inoculation significantly inhibited the transcript levels of *ZIP* in both leaves and roots (Fig. 5). Interestingly, the down-regulated *ZIP* trends in the roots of the DSE-inoculated treatments was consistent with the decreased Cd^2+^ uptake in inoculated maize roots, showing a reduction in root *ZIP* expression by 0.89, 0.74 and 0.59 times for the 10, 50 and 100 mg kg^−1^ Cd treatments, compared with their non-inoculated controls, respectively (Fig. 5).

The elevated Cd stress up-regulated *PCS* expression in both leaves and roots of maize, regardless of inoculated treatment. Additionally, DSE colonization significantly up-regulated *PCS* expression in both leaves and roots of all Cd stress treatments, and in the leaves of the 0 mg kg^−1^ Cd treatment. qPCR data showed *PCS*, encoding proteins involved in phytochelatin biosynthesis that bind free Cd^2+^ in the cytosol, was up-regulated by 1.28, 1.34 and 1.24 times in inoculated maize roots compared to the non-inoculated roots under 10, 50 and 100 mg kg^−1^ Cd exposure, respectively (Fig. 5). *PCS* also had 1.21-, 1.34-, 1.33- and 1.42-fold up-regulation in DSE-inoculated maize leaves than the non-inoculated control under 0, 10, 50 and 100 mg kg^−1^ Cd exposure, respectively (Fig. 5).

With the increasing Cd^2+^ levels, *MTP* expression was up-regulated in the leaves of both DSE-inoculated and non-inoculated maize, with the maximum *MTP* expression observed in maize roots under 50 mg kg^−1^ Cd stress, followed by 100, 10 and then 0 mg kg^−1^ Cd stress. Furthermore, DSE colonization significantly enhanced the transcript levels of *MTP* in leaves by 1.53-, 1.38-, 1.53- and 1.45-fold up-regulation in the DSE-inoculated maize leaves, compared to the non-inoculated plants under 0, 10, 50 and 100 mg kg^−1^ Cd exposure, respectively. In contrast to the leaves, significant DSE-induced up-regulation of *MTP* expression in maize roots was only recorded at 50 and 100 mg kg^−1^ Cd stress (Fig. 5).

## Discussion

Cadmium (Cd), one of the most toxic, non-essential and mobile metallic elements found in soils, is known to be phytotoxic and affect plant physiological processes^20^. In the current study, adverse effects caused by elevated Cd were notable in maize, inoculated with DSE or not. However, *E. pisciphila* colonization significantly alleviated the deleterious effects of excessive Cd amendments and promoted the growth of maize, consistent with our previous study^11^. This suggests that *E. pisciphila*, found colonizing numerous plant roots in an ancient Pb-/Zn- Slag heap in China^21^ and previously reported as a fish-pathogenic fungus^22^,^23^, confers a marked cadmium tolerance to its host plant and plays a functional role as a beneficial plant-associated fungus with DSE morphological characteristics. The reduced Cd accumulation in both shoots and roots of DSE-inoculated maize suggests that plants which accumulate and/or tolerate Cd have evolved an alternative mechanism, utilizing DSE colonization, to resist taking up metals in the soil and/or to tolerate metals inside cells^14^,^19^.

Understandably, excessive Cd accumulated in leaves and roots of both DSE-inoculated and non-inoculated maize with the elevated Cd treatments. Increasingly, data, including the present study, show that accessible toxic Cd alters the levels of metabolic enzymes and indirectly induces oxidative stress by generating reactive oxygen species (ROS), through mechanisms such as lipid peroxidation^24^. The significantly increased MDA content, regarded as a marker for oxidative level, suggests that serious oxidative damage was induced by the elevated Cd stress in both DSE-inoculated and non-inoculated maize. However, the significantly reduced MDA content in the DSE-inoculated treatments, compared to the non-inoculated controls, provides evidence that *E. pisciphila* alleviated oxidative injuries. Subsequently, our data on a battery of antioxidant components, composed of enzymes such as SOD, POD, CAT and the low-molecular weight antioxidants such as glutathione^24^, show that DSE colonization triggered these antioxidant responses to help cope with oxidative stress. In particular, the considerable enhancement of CAT activities in DSE-inoculated plants, at all Cd concentrations, implies the crucial role this enzyme has in DSE-induced plant protection from oxidative stress. Antioxidant properties of the leaves of the DSE-inoculated maize differed from the non-inoculated control, suggesting that DSE fungus may be able to regulate genes encoding for antioxidant enzymes, providing protection against ROS^25^. Similar phenomena have been reviewed from several studies related to mycorrhizal fungal responses^24^. For example, in *Cichorium intybus*, Rozpądek, *et al.*^26^ observed greater activity of SOD and H_2_O_2_ removing enzymes, CAT and POX, in mycorrhizal plants compared to non-mycorrhizal plants, under Cd stress.

The distribution of metallic ions in plant tissues is associated with their toxicity, and selective distribution of toxins is of crucial significance in determining the survival of plants in toxic metal stress^27^. At subcellular levels, Cd can be accumulated in specific cell sites which correspond to less metabolically active areas, e.g. vacuoles or cell wall, where Cd might be insolubly complexed to strongly limit the translocation of Cd^28^. For instance, cell walls, considered a potential reservoir for Cd, accumulated more than half of the cadmium in Cd-tolerant *Kandelia obovata*, while the lowest Cd accumulation was in membranes^29^. Fernández, *et al.*^30^ also concluded that retention of large amounts of Cd in the cell wall was the first strategy in response to metal stress in the Cd accumulator plant, *Dittrichia viscosa*. Interestingly, we observed a distinct subcellular modification of Cd accumulation in both leaves and roots of DSE-inoculated maize, where DSE colonization conferred a greater proportion of Cd accumulated as cell wall integrated/bound-Cd. Thus, a significant reduction of Cd in the other subcellular fractions, especially soluble-Cd, ameliorated the negative effects of excessive Cd accumulation in both leaves and roots of DSE-inoculated maize. Wang, *et al.*^31^ found that colonization by a mycorrhizal fungus (*Glomus intraradices*) altered subcellular distribution and increased the proportion of Cd in the cell wall while reducing the proportion in organelles and membranes. Our data shows that DSE enabled their host plants to have more phytotoxic plasticity via modifications to the composition and structure of their cell wall to facilitate continued cell growth and development while under stress. In fact, increasingly, data show that DSE colonization contributes to host plant nutrition and health, e.g. N and P uptake^16^,^32^. Evidently, cell wall metabolism is strongly dependent on factors such a plant nutritional status, thus the cell wall undergoes compositional variations when grown under varied nutritional availabilities^28^. Such secondary modifications may render cell walls less permeable to Cd, limiting its entry into the cell. Therefore, the enhancement of Cd accumulation in DSE-colonized maize cell wall confers a protective role as both the primary reservoir for Cd, and as an efficient barrier to its penetration in DSE-colonized maize^28^.

Moreover, we noted a distinct shift in the chemical forms of Cd in the DSE-colonized maize. DSE colonization increased the proportion of inactive forms of Cd in maize roots and leaves while reducing the proportion of both soluble and inorganic Cd. Wang, *et al.*^31^ also found that an arbuscular mycorrhizal fungus (*Glomus intraradices*) enabled its host plant (*Medicago sativa*) to convert Cd into inactive forms which resulted in a greater accumulation of inactive forms of Cd in roots and shoots of the mycorrhizal plants than those of non-mycorrhizal plants. We argue that the proportional changes in chemical forms of Cd were of crucial significance for the enhanced tolerance of the DSE plants. In support of this, prevailing evidence suggests that the chemical form of a metal determines its reactivity and solubility. Thus, the speciation of a metal, rather than its total concentration, is the key to understanding its biological functions and toxicities, as this influences its bioavailability^33^. Presumably, DSE-improvement of plant nutrition e.g. N and P uptake, would also contribute to insoluble metal complexation^16^,^32^. A strong body of evidence in the literature has shown that increases in nutritional status are also likely to alleviate stress-related aspects of metal toxicity. For example, Zhang, *et al.*^34^ found Cd integrated into undissolved Cd–phosphate complexes in the cell wall or compartmentalized in the vacuole significantly contributed to the adaptation of the mining ecotypes of *Athyrium wardii* to Cd stress. Under arsenic toxicity, Dixit, *et al.*^35^ also reported that direct, high sulfur supplements contributed to As complexation in rice roots and restricted As translocation to the shoots via processes such as the synthesis of thiolic ligands (non-protein thiols, phytochelatins etc.). Additionally, increasing evidence shows that soil chloride anion, from CdCl_2_·2.5H_2_O used as the Cd supplement in the present study, may affect the phyto-availability of Cd, and cause changes to plant growth and Cd accumulation^36^. Therefore, when interpreting our results across Cd treatments one must take into account that the observed effects could also be influenced by chloride anion concentrations.

Subcellular repartitioning of Cd in DSE-inoculated maize suggests that DSEs tightly regulate the processes of both essential nutrition and non-essential metal accumulation. In the current study, gene expression patterns showed that three maize genes which are involved in metal uptake, translocation and chelation, were strictly regulated by both DSE-colonization and Cd stress. In general, it is believed that Cd^2+^ enters root cells via transporters for nutritional ions, for example the *ZIP* gene family, transporting Fe^2+^ and Zn^2+^ 37. Our results show that DSE-inoculation significantly down-regulated *ZIP* in both roots and leaves of all treatments except the low Cd treatment leaves; this suggests that the observed reduction in Cd uptake by DSE-inoculated maize roots, and the restricted translocation of Cd from roots to leaves was consistent with maize *ZIP* genes being involved in non-essential Cd uptake and translocation^38^. In contrast to the down-regulated *ZIP*, DSE inoculation significantly up-regulated metal tolerance protein encoding gene *MTP*, one of the most important Cd vacuolar sequestration transporters in plants^39^, in both roots and leaves. Migocka, *et al.*^40^ also confirmed that *CsMTP1* and *CsMTP4* from cucumber, homologous to MTP transporters, were able to mediate the hypersensitivity of mutant strains to Zn and Cd through the increased sequestration of metals within vacuoles. Thus, we argue that the up-regulated *MTP* would contribute to the detoxification of toxic Cd concentrations through sequestration to specific cellular compartments, such as vacuoles. In addition, enhancement of *PCS* production, a phytochelatin synthase gene, would improve the catalyzed transpeptidation of glutathione and related thiol peptides^41^, which might play fundamental roles in Cd^2+^ transport and detoxification^42^. Interestingly, the DSE-induced regulation of the *ZIP, MTP* and *PCS* genes, in the present study, was consistent with the observed changes in subcellular repartitioning and chemical forms of Cd in DSE-inoculated maize. But, one should keep in mind that global gene expression profiling of maize responses to elevated Cd stress under DSE inoculation is in need of further attention.

In conclusion, inoculation of energy maize roots with *E. pisciphila*, a potential DSE plant symbiont, indeed resulted in a marked tolerance to Cd with a significant decrease in cadmium phytotoxicity and a significant increase in both maize root and shoot growth. The combination of plant growth enhancement and reduced metal translocation, caused by microbial inoculation, could be a promising strategy for enabling profitable, yet safer crop production in Cd contaminated arable soils. Our results illustrate that the DSE, *E. pisciphila* enhanced plant tolerance to Cd through the combined mechanisms of altering both the chemical forms and distribution of Cd among different plant tissues, and by triggering antioxidant systems together with tightly regulated modifications to maize gene expression; this will help shed light on the alternative resistance mechanisms of host plants bioaugmented by DSE colonization, and on how to improve the plant strategies used to tolerate Cd. However, one must keep in mind that a complex and cooperative regulatory network controls intracellular ion levels, including both essential nutrition and non-essential metals^43^,^44^. Additionally, more emphasis should be placed on the DSE-enhanced roles of macro- and micro-nutrients (S, Si, P, etc.) in Cd accumulation/tolerance, because these nutrients may have the potential to enhance Cd accumulation capacity in plants^45^,^46^,^47^.

## Materials and Methods

### Soil and biological materials, and experimental design

*Exophiala pisciphila* H93, a dominant root-associated DSE fungus, was isolated from the roots of revegetation plants naturally growing in an ancient Pb-/Zn- slag heap in Huize county, Yunnan province, southwest China^21^. *E. pisciphila* H93 (accession number ACCC32496, Agricultural Culture Collection of China) was selected as a DSE inoculant due to its previously examined beneficial functions in symbiosis and extreme tolerance to metal ions^11^,^48^, which dominantly colonize the roots of revegetation plants naturally growing in an ancient Pb-/Zn- slag heap in Huize county, Yunnan province, southwest China^21^. Huidan No. 4, a native maize cultivar in Yunnan province, China, was selected as host plant in the present investigation.

Huidan No. 4 seeds were surface-sterilized by immersion in 75% ethanol for 10 min and then in 10% sodium hypochlorite for 10 min. Sterilized seeds were then thoroughly rinsed with sterile water and aseptically germinated on water agar medium (8 g L^−1^ agar) at 25 °C. Three days later, the germinated seeds were transplanted into 250 mL Erlenmeyer flasks (one plant per flask) containing 300 g sterile silica sand culture substrate (Zhiyuan Reagent Co., Ltd, Tianjin, China; autoclaved for 2 h at 121 °C three times with 2-day intervals) supplemented with a series of Cd^2+^ treatments (0, 10, 50, 100 mg kg^−1^ Cd^2+^; CdCl_2_·2.5H_2_O), consistent with previous research^11^. The substrate was then inoculated with either two fungal disks (Φ 0.5 cm) cut from a 14-day-old *E. pisciphila* H93 PDA culture or, for a control treatment, two autoclaved fungal disks (Φ 0.5 cm). The eight treatments (+/− *E. pisciphila* H93 × 4 [Cd^2+^]) were each replicated eight times resulting in a total of sixty-four maize seedlings. The flasks were placed in a phytotron with a 14 h photoperiod (30, 000 lx) at 25/18 °C (day/night) and 75% humidity for 30 days. At the start of the growth period the flasks were watered thoroughly with 100 mL sterile 1/2 Hoagland’s solution^49^ and then, after 10 days, all flasks received 10 mL of 1/2 Hoagland’s every two days.

After 30 days, the root and shoot (leaf + stem) tissue was harvested separately for all treatments. The samples were thoroughly washed with deionized water and one root and leaf subsample from each seedling was immediately placed in −80 °C, for subsequent analyses, while the remaining tissue was dried (80 °C, 48 h) to constant weight and weighed. DSE fungal colonization intensity was determined using the gridline intersect method with 200 to 300 intersects^50^. The dried samples were then digested with HNO_3_-HClO_4_ (3:1, v:v) and Cd concentration was determined using an atomic absorption spectrophotometer (Varian AA240FS, USA), as described previously^11^.

### Antioxidant enzyme and antioxidant determination

Frozen root and leaf tissue was ground with a mortar and pestle in chilled homogenization buffer solution containing 50 mM phosphate buffer (pH 7.0). The homogenate was centrifuged at 12 000 g for 10 min at 4 °C and the supernatant was collected for assaying antioxidant enzyme activity and content. As described previously^51^,^52^,^53^,^54^, the activities of antioxidant enzymes, including total superoxide dismutase (SOD), catalase (CAT), peroxidase (POD) and the content of soluble proteins and glutathione (GSH) were assessed. Additionally, malondialdehyde (MDA), one of the oxidative stress decomposition products of polyunsaturated membrane fatty acids, was also evaluated^55^. All assays were conducted using commercial chemical assay kits (Nanjing Jiancheng Bioengineering Institute, China) according to the manufacturer’s protocol and each sample was duplicated three times.

### Distribution of cadmium in subcellular fractions of leaves and roots

Following the method described by Wu, *et al.*^56^, subcellular tissue fractions of each sample were extracted as follows; 0.5 g of leaf or root tissue was homogenized in cold extracting buffer containing 50 mM Tris–HCl, 250 mM sucrose, 1.0 mM DTE (C_4_H_10_O_2_S_2_), 5.0 mM ascorbic acid and 1.0% w:v Polyclar AT PVPP (pH 7.5) with a chilled mortar and pestle. The homogenate was then sieved through a nylon cloth (240 μm) and cell wall residue on the nylon cloth was washed three times with the buffer. The filtrate was then centrifuged at l 500 g for 10 min (leaf filtrate), or 2 500 g for 20 min (root filtrate), and the pellet obtained was the chloroplast or trophoplast fraction, for leaf and root respectively. Subsequently, the supernatant was centrifuged at 15 000 g for 35 min and the pellet was referred to as the membrane and organelle fraction, while the supernatant was labelled as the soluble fraction. All steps were performed at 4 °C and then Cd content of subcellular fractions of leaves and roots was determined via spectrophotometry, as described above.

### Extraction of Cd in different chemical forms

Cd, associated with different chemical forms in the maize and culture substrate, was extracted with previously established solutions^57^. From each flask, 0.5 g frozen rhizosphere sand substrate, leaf and root tissue were homogenized in 50 mL of their respective extraction solutions with a chilled mortar and pestle. The tissue homogenate was incubated at 180 rpm for 22 h at 25 °C, and then centrifuged at 5 000 g for 10 min to obtain the first supernatant solution. Then, residual sediment was twice re-suspended in 50 mL of extraction solution, incubated for 2 h, and centrifuged at 5 000 g for 10 min. Finally, the supernatants from all three centrifugation steps were pooled, and Cd concentrations in various chemical forms were measured, which was extracted with the following 5 different extraction solutions, in order:

1. 80% ethanol, extracting inorganic Cd giving priority to nitrate/nitrite, chloride, and aminophenol Cd.
2. Deionized water (dd-H_2_O), extracting water-soluble Cd-organic acid complexes, and Cd(H_2_PO_4_)_2_.
3. 1 M NaCl, extracting pectates and protein integrated Cd.
4. 2% Acetic acid (HAC), extracting undissolved Cd phosphate including CdHPO_4_ and Cd_3_(PO_4_)_2_.
5. 0.6 M HCl, extracting Cd oxalate.

### Gene expression of *ZIP, PCS, MTP*

For total RNA extraction, approximately 0.3 g fresh maize leaf and root tissue from all 8 treatments was collected, immediately frozen, ground in liquid nitrogen, and homogenized in RNAiso Plus solution. Total RNA was extracted using the RNAiso Plus Kit (TaKaRa, Japan), according to the manufacturer’s protocol. Then the quantity and the purity of the RNA were determined by UV measurement using a NanoDrop 2000c spectrophotometer (Thermo Scientific, Loughborough, UK). Complementary DNA was synthesized from the total RNA using the PrimeScript RT reagent kit with gDNA eraser (TaKaRa, Japan). The synthesized cDNA was used as the template for quantitative PCR (qPCR). Specific primer pairs of 3 genes encoding for Zinc transporter SLC39A7 isoform 1 (*ZIP*, GenBank No. NM_001137726; Forward:5′-CCT CTC TGC GTT GGT TGC TCT-3′, Reverse: 5′-TTG ATG GTT GTT TTC TGG TCG T-3′), phytochelatin synthetase-like protein (*PCS*, GenBank No. AF160475; Forward: 5′-CGG CAA TGT TCT GGG GTG TA-3′, Reverse: 5′-CGA AAG TGA AAG TCC GGG AGT C-3′), and metal tolerance protein A2 (*MTP*, GenBank No. EU961941; Forward: 5′CCA CTC AGG CAC TGG ACA A-3′, Reverse: 5′-CCC CAA GCA CAT GAA GGT AA-3′) were designed. The 18S rRNA gene (GenBank No. AF168884) was amplified by a primer pair (Forward: 5′-CCA TCC CTC CGT AGT TAG CTT CT-3′, Reverse: 5′-CCT GTC GGC CAA GGC TAT ATA C-3′) and used as a reference gene to normalize the quantification gene expression. To assess the interference of DSE RNA in the DSE-colonized roots samples, negative qPCR control was carried out to evaluate the specificity of the primers for maize *ZIP, PCS, MTP* and *18S rRNA* genes using DSE cDNA, extracted from DSE culture under EC_50_ Cd stress *in vitro*. No PCR products were observed from the DSE cDNA samples, suggesting that the specificity of our primers excluded interference from DSE RNA in our experimental samples.

qPCR was performed using SYBR Premix Ex Taq (TaKaRa, Japan), and qPCR reactions were conducted using the Applied Biosystems 7500 Real-Time PCR System (Applied Biosystems, CA, USA) according to the manufacturer’s instructions. All of the reaction mixtures were heated at 95 °C for 30 s and subjected to 40 PCR cycles of 95 °C for 5 s and 60 °C for 34 s, and the resulting fluorescence was measured. PCR reactions without cDNA template were used as negative controls. All of the reactions were performed with three biological replicates, and technical replication of each biological replicate was conducted independently three times. Compared to the non-inoculated maize gene expression under no Cd exposure, the relative gene expression quantity of the three genes was determined using the Applied Biosystems 7500 Real-Time PCR System and data were analyzed using the 2^−ΔΔCt^ method as described by Winer, *et al.*^58^.

### Statistical analysis

Data on subcellular distribution and chemical forms of Cd in maize inoculated with DSE (+DSE) or not (-DSE) under each Cd^2+^ stress was analyzed using an ANOVA. Data on Cd concentrations, antioxidant enzyme activities and antioxidant contents, and gene expression were analyzed using an ANOVA with a post-hoc Tukey’s HSD test. Before ANOVA analyses, all data for all treatment groups were verified to meet the assumptions of normality and homogeneity of variances by Shapiro–Wilk and Levene tests, respectively. All analyses were performed using SPSS 16.0 software package (SPSS Inc., Chicago, IL, USA). The rejection level was set at *α* < 0.05. Unless stated otherwise, all values were reported as means and standard errors of means (SEM, *n* ≥ 8).

## Additional Information

**How to cite this article**: Wang, J.-l. *et al.* Unraveling the role of dark septate endophyte (DSE) colonizing maize (*Zea mays*) under cadmium stress: physiological, cytological and genic aspects. *Sci. Rep.* **6**, 22028; doi: 10.1038/srep22028 (2016).

## Figures and Tables

**Table 1:** 

Maize	Cd stress (mg kg^−1^)	Inoculation	Cd concentrations in each cell fraction (mg kg^−1^)			
			Cell wall	Chloroplast-leaf/trophoplast-root	Membrane and organelle	Soluble
Root	10	−DSE	65.77 ± 1.16	22.12 ± 1.05	7.57 ± 0.55	58.35 ± 3.03
		+DSE	34.33 ± 0.95***	4.62 ± 0.46***	1.65 ± 0.39***	25.58 ± 1.10***
	50	−DSE	212.98 ± 6.60	120.97 ± 1.83	33.78 ± 0.81	234.45 ± 4.35
		+DSE	126.45 ± 3.03***	25.60 ± 1.98***	13.48 ± 1.27***	83.67 ± 0.52***
	100	−DSE	839.63 ± 4.87	363.06 ± 9.04	245.82 ± 13.09	809.57 ± 12.71
		+DSE	679.56 ± 9.59***	98.33 ± 6.14***	72.77 ± 1.90***	367.15 ± 9.31***
Leaf	10	−DSE	21.19 ± 0.01	11.65 ± 0.85	0.73 ± 0.03	25.13 ± 2.13
		+DSE	17.63 ± 0.88**	6.18 ± 0.95**	0.63 ± 0.03*	11.68±1.83**
	50	−DSE	70.78 ± 3.05	36.33 ± 1.10	9.10 ± 0.53	76.55 ± 2.20
		+DSE	66.88 ± 2.03	23.38 ± 1.12***	5.85 ± 0.44**	32.28 ± 1.23***
	100	−DSE	268.68 ± 5.60	156.40 ± 2.85	63.20 ± 0.35	206.95 ± 8.41
		+DSE	153.68 ± 2.78***	74.72 ± 1.30***	18.67 ± 1.10***	54.37 ± 2.82***

Subcellular distribution of Cd in maize inoculated with DSE (+DSE) or not (−DSE) under a series of Cd^2+^ stresses (0, 10, 50, 100 mg kg^−1^), examining the effect of DSE inoculation (mg kg^−1^, means ± SEM, *n* = 6; *indicates significant differences at 0.01 < *p* < 0.05, **indicates significant differences at 0.001 < *p* < 0.01, ***indicates significant differences at *p* < 0.001 level, ANOVA). No Cd^2+^ was observed for the no Cd control (data not shown).

**Table 2:** 

Material	Cd stress (mg kg^−1^)	Inoculation	F_ethanol_	F_dd-H2O_	F_NaCl_	F_HAc_	F_HCl_	F_residue_
Matrix	10	−DSE	0.09 ± 0.01	0.28 ± 0.01	3.71 ± 0.04	3.21 ± 0.02	0.07 ± 0.01	1.86 ± 0.10
		+DSE	0.11 ± 0.00**	0.32 ± 0.01**	3.94 ± 0.01**	3.34 ± 0.01**	0.11 ± 0.01**	1.75 ± 0.04
	50	−DSE	0.94 ± 0.03	1.64 ± 0.08	21.88 ± 0.12	10.98 ± 0.03	0.69 ± 0.02	13.43 ± 0.07
		+DSE	1.05 ± 0.01**	1.85 ± 0.07*	23.06 ± 0.01***	12.16 ± 0.02***	0.80 ± 0.01**	10.99 ± 0.02***
	100	−DSE	16.76 ± 0.16	7.05 ± 0.10	26.86 ± 0.03	14.33 ± 0.19	4.17 ± 0.16	28.87 ± 0.57
		+DSE	14.43 ± 0.09*	4.26 ± 0.11*	34.49 ± 1.35**	18.70 ± 0.32*	1.75 ± 0.16***	25.57 ± 0.89*
Root	10	−DSE	3.63 ± 0.11	24.97 ± 0.21	32.30 ± 0.52	10.60 ± 0.10	4.51 ± 0.11	19.33 ± 0.09
		+DSE	2.63 ± 0.05***	9.83 ± 0.12***	31.02 ± 0.81	20.20 ± 0.92***	7.80 ± 0.03***	11.64 ± 0.04***
	50	−DSE	11.76 ± 0.11	92.43 ± 0.21	105.88 ± 0.52	41.48 ± 0.10	13.51 ± 0.11	71.06 ± 0.09
		+DSE	8.87 ± 0.05***	15.62 ± 0.12***	122.97 ± 0.81***	82.03 ± 0.92***	35.94 ± 0.03***	32.10 ± 0.04***
	100	−DSE	132.91 ± 1.20	1109.17 ± 1.02	1225.72 ± 2.65	501.70 ± 3.51	89.80 ± 1.85	931.96 ± 3.67
		+DSE	105.52 ± 1.48***	539.12 ± 1.57***	1279.26 ± 2.63***	850.49 ± 1.51***	199.52 ± 1.63***	741.62 ± 3.01***
Leaf	10	−DSE	7.67 ± 0.37	12.22 ± 0.87	16.26 ± 0.35	7.69 ± 0.45	6.67 ± 0.68	12.70 ± 0.91
		+DSE	3.01 ± 0.11***	13.31 ± 0.82	13.99 ± 1.09*	9.56 ± 0.48**	7.55 ± 0.87	9.84 ± 0.70*
	50	−DSE	25.03 ± 0.24	36.12 ± 0.34	46.48 ± 0.99	21.02 ± 0.98	17.58 ± 1.04	51.78 ± 0.94
		+DSE	12.21 ± 0.79***	32.86 ± 0.91**	31.12 ± 1.02***	27.03 ± 0.55**	22.42 ± 0.94**	31.34 ± 0.66***
	100	−DSE	85.87 ± 1.95	152.28 ± 1.68	195.47 ± 2.53	60.94 ± 3.12	61.61 ± 3.62	192.48 ± 1.89
		+DSE	23.90 ± 0.64***	69.55 ± 3.37***	86.10 ± 2.34***	80.77 ± 4.35**	59.73 ± 0.83	61.7 ± 3.67***

Distribution of Cd chemical forms by 5 different extraction solutions (F_ethanol_ represents inorganic Cd extracted by 80% ethanol, F_dd-H2O_ for water soluble Cd by dd-H_2_O, F_NaCl_ for pectate and protein Cd by NaCl, F_HAc_ for undissolved Cd by acetic acid, F_HCl_ for Oxalate Cd by HCl, F_residue_ for residue Cd) as described by Fu, *et al.*^57^ in the culture matrixes and maize roots and leaves, under a series of Cd stresses (0, 10, 50, 100 mg kg^−1^), examining the effect of DSE inoculation (mg kg^−1^, means ±  SEM, *n* = 6; *indicates significant differences at 0.01 < *p* < 0.05, **indicates significant differences at 0.001 < *p* < 0.01, ***indicates significant differences at *p* < 0.001 level, ANOVA). No Cd^2+^ was observed for the no Cd control (data not shown).
